# Insights into Transcriptomes of Big and Low Sagebrush

**DOI:** 10.1371/journal.pone.0127593

**Published:** 2015-05-28

**Authors:** Mark D. Huynh, Justin T. Page, Bryce A. Richardson, Joshua A. Udall

**Affiliations:** 1 Plant and Wildlife Science Department, Brigham Young University, Provo, UT, 84602, United States of America; 2 Rocky Mountain Research Station, USDA Forest Service, Provo, UT, 84606, United States of America; University of North Carolina at Charlotte, UNITED STATES

## Abstract

We report the sequencing and assembly of three transcriptomes from Big (*Artemisia tridentata *ssp. *wyomingensis and A*. *tridentata *ssp. *tridentata*) and Low (*A*. *arbuscula* ssp. *arbuscula*) sagebrush. The sequence reads are available in the Sequence Read Archive of NCBI. We demonstrate the utilities of these transcriptomes for gene discovery and phylogenomic analysis. An assembly of 61,883 transcripts followed by transcript identification by the program TRAPID revealed 16 transcripts directly related to terpene synthases, proteins critical to the production of multiple secondary metabolites in sagebrush. A putative terpene synthase was identified in two of our sagebrush samples. Using paralogs with synonymous mutations we reconstructed an evolutionary time line of ancient genome duplications. By applying a constant mutation rate to the data we estimate that these three ancient duplications occurred about 18, 34 and 60 million years ago. These transcriptomes offer a foundation for future studies of sagebrush, including inferences in chemical defense and the identification of species and subspecies of sagebrush for restoration and preservation of the threatened sage-grouse.

## Introduction

The sagebrushes (*Artemisia* subgenus *Tridentatae*) are pivotal members and the most abundant and widespread vegetation of the semi-arid ecosystems of western North America. Sagebrush ecosystems cover vast areas of the western United States and Canada [1]. This study focuses on two species of subgenus *Tridentatae*: big sagebrush (*Artemisia tridentata)* and low sagebrush (*A*. *arbuscula* ssp. *arbuscula)*. *A*. *tridentata* occupies about 43 million ha of the United States and includes three major subspecies: *A*. *tridentata* ssp. *tridentata* and *A*. *tridentata* ssp. *vaseyana* exist as both diploids and tetraploids, while *A*. *tridentata* ssp. *wyomingensis* is exclusively tetraploid. In comparison, *A*. *arbuscula* occupies about 28 million ha of the United States with four described subspecies, including diploid, tetraploid and occasionally hexaploid cytotypes [2], [3]. Low sagebrush typically occupies ridgelines and uplands with shallow soils, whereas big sagebrush occupies deeper soils [4], [5], [6]. The two species can have parapatric occurrences, especially between *A*. *arbuscula* and *A*. *tridentata* ssp. *vaseyana*.

The sagebrush ecosystems are habitat and forage for numerous sagebrush-dependent wildlife species. Most notably is the greater sage-rouse (*Centrocercus urophasianus*), which is of conservation concern due to a declining habitat and shrinking breeding populations. Since being listed in 2010 by the U.S. Fish and Wildlife Service as a candidate for the endangered species list, it is currently the subject of one of the largest conservation efforts in North America [7]. Sage-grouse eat sagebrush leaves exclusively in winter months and they remain a primary food source throughout the rest of the year. For sage-grouse, habitat selection and forage of sagebrush is guided in part by avoidance of plants with higher concentrations of monoterpenes [8]. Sage-grouse may be especially sensitive to terpenes when selecting a food source because they may lack the ability to volatize ingested terpenes through mastication [9] or metabolize these compounds prior to absorption as in some ruminants [10], [11]. A greater understanding of the chemical components that affect sagebrush palatability is a critical goal for sage-grouse conservation. For example, when available, sage-grouse prefer *A*. *nova* and then *A*. *wyomingensis* compared to other species of sagebrush, a preference that is proposed to be correlated with decreasing leaf terpene concentration [6], [12]. This discrimination appears to be deeper than selecting solely between species. Indeed, Frye et al. [8] demonstrated that feeding selection within a conspecific patch of sagebrush is specific to plants with lower monoterpenes, regardless of species. Feeding preference based on terpene concentration is not unique to sage-grouse. Pygmy rabbits have also shown sensitivity to terpene concentration in a natural setting [13]. Because conservation will largely be based on restoration at the ecosystems level, a finely tailored effort is needed that considers both the types of terpenes produced and their expression profiles among and within species.

Terpenoids have also been shown to be important in inter- and intraspecies plant communication [14]. Plant volatiles, including terpenes, released from the leaves of injured sagebrush plants function in priming the defense of the surrounding plant community [15]. As a result of this functional versatility, a large amount of research has been geared towards the isolation and classification of terpenes produced by the genus *Artemisia* (see review by Turi et al. [16]), however, the identification of genes and alleles involved in terpene biosynthesis and differences among sagebrush species or populations has not been reported.

While much work has been done in characterizing sagebrush based on taxonomic characters and cytology, little has been done to describe the transcriptomes of sagebrush. An NCBI search for *Artemisia* nucleotide sequences returns 26 sequences for *A*. *arbuscula* and less than 600 sequences for *A*. *tridentata*. The only transcriptome study of sagebrush was reported by Bajgain et al. [17], where they identified single nucleotide polymorphism (SNP) data from transcript amplicons of three big sagebrush subspecies in an attempt to elucidate complex polyploid and hybrid relationships. However, the combined Illumina and 454 sequencing technologies used in the study may not have fully sampled the transcriptome. Here we attempt to more fully sample the transcriptome with deeper sequencing and by including more than one species of sagebrush. This data not only provides the basis for elucidating specific biosynthetic pathways, but they also enable the study of gene duplication. Gene duplication drives plant evolution by creating duplicate genes that can increase the levels of specific gene products or mutate to acquire specialized or completely novel functions [1], [18]. In addition to single gene duplications, whole-genome duplications (WGD) may occur. WGD are thought to drive evolution by creating a larger background for mutation that, in some ecological circumstances, may lead to a greater survivability of polyploids [3]. These single gene duplications and WGD can be detected by the proxy use of synonymous mutations [19], [20]. The chronology of these duplications provides inferences about the origin of a particular species and divergent taxa.

In this paper, we present the assembly of three transcriptomes representing two species of subgenus *Tridentatae*. We utilize the transcriptomes to identify and analyze a putative ortholog of a terpene synthase (TS) present in both *A*. *tridentata* and *A*. *arbuscula* and for detecting ancient duplication events using synonymous mutation rates between paralogs. These transcriptomes will undoubtedly be useful for further elucidating the complex evolutionary history of sagebrush through transcript identification and SNP detection. They may also serve as reference transcriptomes for subsequent transcriptome analyses within the genus and for gene expression analyses (RNASeq experiments).

## Materials and Methods

### RNA Sequencing

Five half-sib seedlings from each *A*. *tridentata* ssp. *wyomingensis* (UTW1, 38.3279 N, 109.4352 W) *A*. *tridentata* ssp. *tridentata* (UTT2, 38.3060 N, 109.3876 W) and *A*. *arbuscula* ssp. *arbuscula* (CAV-1, 40.5049 N, 120.5617 W) were grown simultaneously in a petri dish on top of wetted filter paper for two days. No specific permissions were required for these locations and none of the species are endangered or protected. Seedlings were then flash frozen in liquid nitrogen and ground using a mortar and pestle. RNA was extracted using a Norgen RNA Purification Kit (Norgen Biotek Corp., Ontario, Canada). Sequencing libraries were prepared using an Illumina Tru-seq RNA Kit V2 (Illumina Inc., San Diego, California). Libraries were then pooled and multiplexed on an Illumina MiSeq lane and sequenced as 250 bp paired-end reads at the Center for Genome Research and Biocomputing, Oregon State University.

### Transcriptome Assembly

Illumina reads were trimmed for quality using default settings in the program Sickle (github.com/najoshi/sickle). Reads were then assembled using the program SOAPdenovo-trans [21] at variable k-mer lengths ranging from 35 to 127 in increments of 4. The best assembly for each read set was based on N50 and the number of scaffolds. Other modified parameters included the number of scaffolds > 800 base pairs (bp) and the number of bp in scaffolds > 800 bp.

### Transcript Characterization

Assembled transcriptomes were uploaded to the program TRAPID [22] where transcripts could be identified by protein domains related to terpene synthases (IPR005630). Transcripts were then blasted on NCBI using blastx [23] with the NR database for putative orthologs. To compare the different sagebrush groups, a three-way blast was also performed using a custom script to identify orthologs between sagebrush samples. The default settings in Geneious version 6.05 (Biomatters Ltd., Auckland, New Zealand) were used to align and call SNPs between putative orthologs.

### Ancient Gene Duplication Detection

Because of its greater depth of coverage, paralogs in *A*. *arbuscula* were detected by a self-blast with a maximum e-value threshold of 1e^-20^. Reciprocal blast hits were considered as potential paralogs. The synonymous mutation rate (Ks) was calculated for each paralog pair. A histogram of pairwise of Ks values was plotted. The highest peak was taken as the best estimate of a duplication event. We then calculated the time of this event by using the estimated background mutation rate in dicots used by Blanc and Wolfe [19] of 1.5 x 10^–8^ substitutions per synonymous site per year. The location and number of peaks were detected using the program EMMIX [24] by selecting the model with lowest Bayesian information criterion from models predicting 1–10 peaks. Statistically significantly peaks were identified using SiZer [25] a program that determines peak significance (p<0.05) by detecting changes in the slope of a curve.

## Results

### Transcriptome Assembly

**Fig 1:**
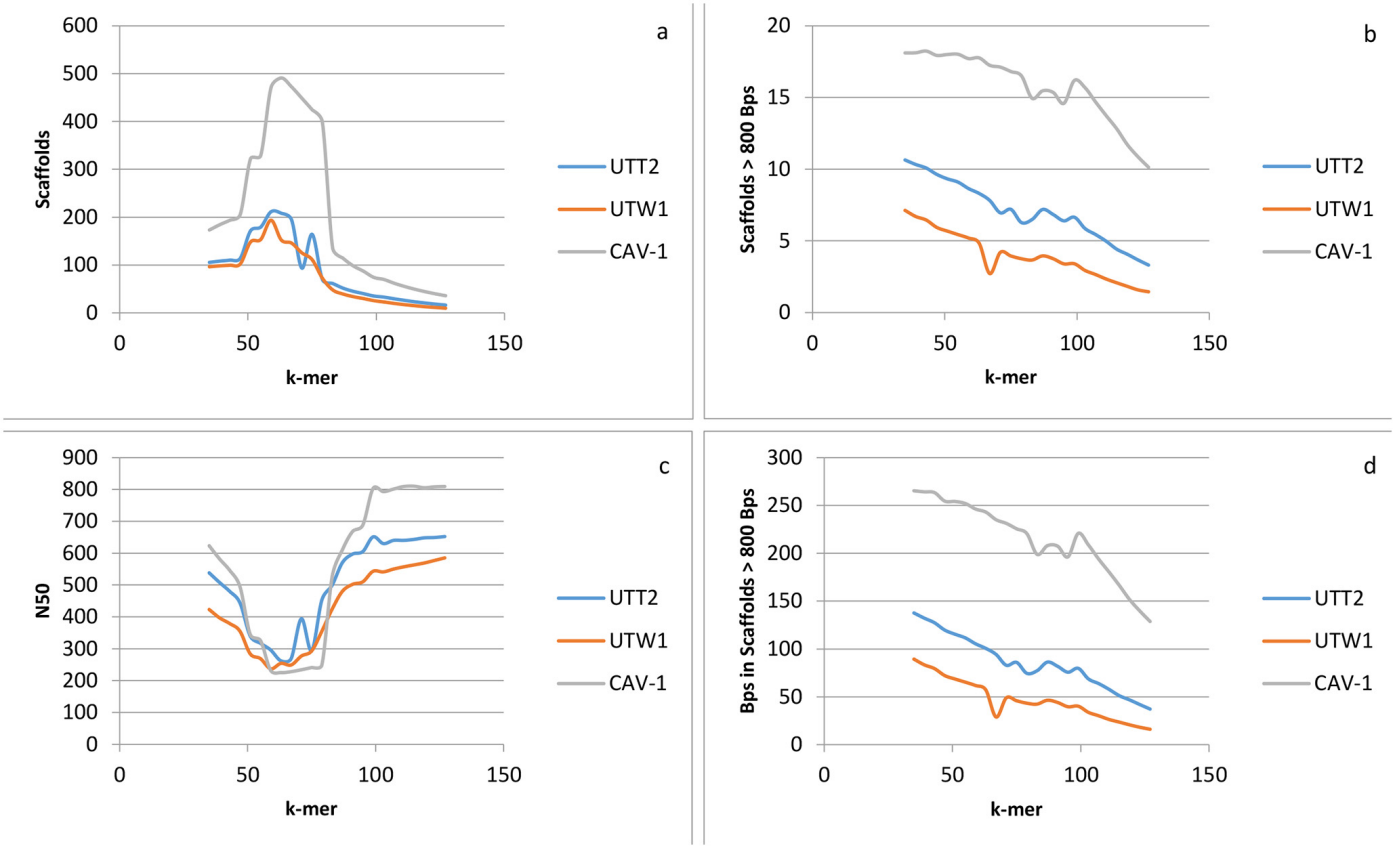
Assembly statistics based on variable k-mer lengths. Transcript assemblies based on variable k-mer lengths ranging from 35–127 in multiples of four of *A*. *tridentata tridentata* (UTT2), *A*. *tridentata wyomingensis* (UTW1) and *A*. *arbuscula* (CAV-1). a) Thousands of scaffolds vs. k-mer length. b) Thousands of scaffolds > 800 bp vs. k-mer length. Scaffolds are divided by 1000. c) N50 vs. k-mer length. d) Millions of scaffolds > 800 vs. k-mer length.

Trimmed 250 bp paired-end reads were assembled *de novo* using SOAPdenovo-Trans at variable k-mer lengths for a total of 35 assemblies for each sagebrush sample. The best assembly was chosen based on number of scaffolds, number of scaffolds >800 bp, number of bp in scaffolds > 800 bp, and N50 (Fig 1). At short k-mer lengths (~35–47), the assembler was not able to sufficiently differentiate similar sequences, so they collapsed together. At moderate k-mer lengths (~47–75), contigs were again broken—likely due to bubbles, assemblies split by polymorphisms, in the contig graph. At long k-mer lengths (~75–127), the assembler was able to differentiate similar regions and make a less error-prone assembly. In all cases, the best assembly for all samples was with a k-mer length of 127. A larger k-mer length may have produced a more acceptable assembly; however, SOAPdenovo-Trans is currently limited to 127-mers. Assemblies of 127-mers had the least amount of scaffolds coupled with the greatest N50. A smaller number of scaffolds with a greater N50 indicate that the assembler was able to join together multiple scaffolds as contigs. This is also indicated by the decreasing number of scaffolds > 800 bps and the number of bps in scaffolds > 800 bp. The assemblies with highest quality are summarized in Table 1.

**Table 1:** Summary of 127 k-mer assemblies using SOAPdenovo-trans.

	UTT2	UTW1	CAV-1
**Scaffolds**	16276	9741	35866
**Bps in Scaffolds > 800 bps**	3720411	1612837	12873716
**# of Scaffolds > 800 bps**	3310	1448	10131
**N50 Scaffold Length**	652	585	809

The number of scaffolds, bps in scaffolds > 800, scaffolds > 800 bps and N50 length for 2 species of sagebrush: *A*. *tridentata tridentata* (UTT2), *A*. *tridentata wyomingensis* (UTW1) and *A*. *arbuscula* (CAV-1).

### Transcriptome Characterization

**Fig 2:**
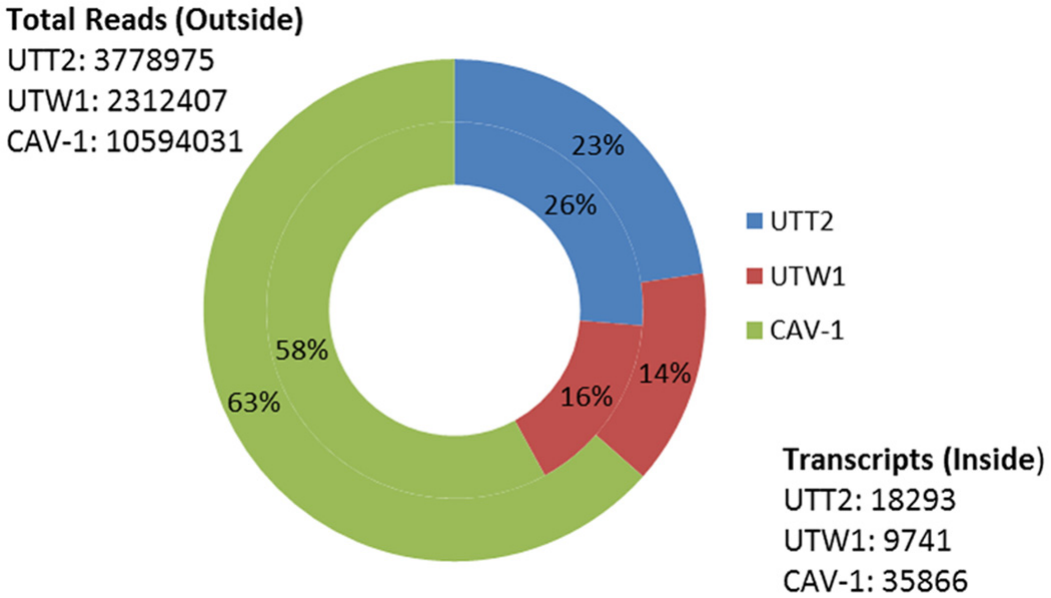
Distributions of detected reads and transcripts. The outside ring represents the number of initial reads and the inside ring represents the number of detected transcripts for *A*. *tridentata tridentata* (UTT2), *A*. *tridentata wyomingensis* (UTW1) and *A*. *arbuscula* (CAV-1). Reflecting the number of reads, the majority of detected transcripts were from CAV-1, while both UTT2 and UTW1 had a similar number of reads between them.

The program TRAPID identified a total of 61,883 transcripts, representing 3427 GO terms and a total of 6,067 gene families with the greatest number of transcripts (407) mapping to the 568_HOM000025 gene family, which is associated with ATP-binding. The transcripts are divided unevenly between the samples with the majority of transcripts detected in *A*. *arbuscula*, likely because of the increased read coverage from that sample (Fig 2). More *A*. *tridentata* transcripts would likely be discovered with increasing read coverage.

### Terpene Synthases

**Fig 3:**
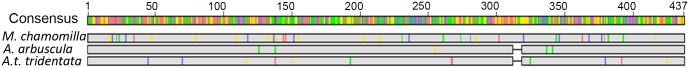
Multiple sequence alignment for a partial transcript of the MrTPS5 gene. A multiple sequence alignment of *A*. *arbuscula*, *A*. *tridentata* and *M*. *chamomile* showing the same 6 SNP base pair deletions present in the genus Artemisia. SNP loci are highlighted as blue for cytosine, green for thymine, yellow for guanidine and red for adenine. Geneious generated the consensus sequence by a majority vote consensus.

As an example of how these transcriptomes may be used, 16 transcripts related to terpene synthases (TS) were found by searching for protein domains associated with terpene synthases (IPR001906) or by the gene family HOM000066. The 16 transcripts—12 in *A*. *arbuscula*, 3 in *A*. *t*. ssp. *tridentata* and 1 in *A*. *t*. ssp. *wyomingensis*—are listed in S1 Table. Blasting transcript C44821 from *A*. *arbuscula* against the NR database showed a single match of 89% percent identity with an E-value of 1e^-255^ and query coverage of 100% for terpene synthase 5 (TPS5) identified in chamomile (*Matricaria chamomilla*). The putative TPS5 of *A*. *arbuscula* ssp. *arbuscula* was used to search the transcriptomes of *A*. *tridentata* ssp. *wyomingensis* and *A*. *tridentata* ssp. *tridentata*. A single hit was found for *A*. *tridentata* ssp. *tridentata* (C12295) with an E-value 1e^-255^ and 96% identity. A multiple alignment (Fig 3) of the three transcripts revealed 38 SNP loci in chamomile, 12 SNP loci in big sagebrush and 5 SNP loci in low sagebrush compared to the consensus sequence of all three sequences. Both sagebrush species also possessed 2 tandem amino acid deletions when compared to chamomile. There were 19 shared non-synonymous mutations in both the sagebrushes. *A*. *arbuscula* ssp. *arbuscula* and *A*. *tridentata* had 5 and 9 unique non-synonymous sites, respectively. *A*. *annua* currently represents most of the available transcript data for the genus *Artemisia* on NCBI. Though *A*. *annua* is classified under the same genus, it is not a sagebrush and despite having the largest collection of published sequences for genus *Artemisia* we could not find an orthologous sequence for this putative TPS5.

### Detection of Ancient Gene Duplication

Excluding self-hits and hits that were too divergent for the Jukes-Cantor model of DNA substitution, we detected 4383 viable paralog hits for peak detection. The maximum detected Ks value was 1.4640 and the minimum was 0.0011 with a median valued of 0.2062. We deliberately included multiple potential paralogs for each sequence in order to accurately detect historic genome duplications.

**Fig 4:**
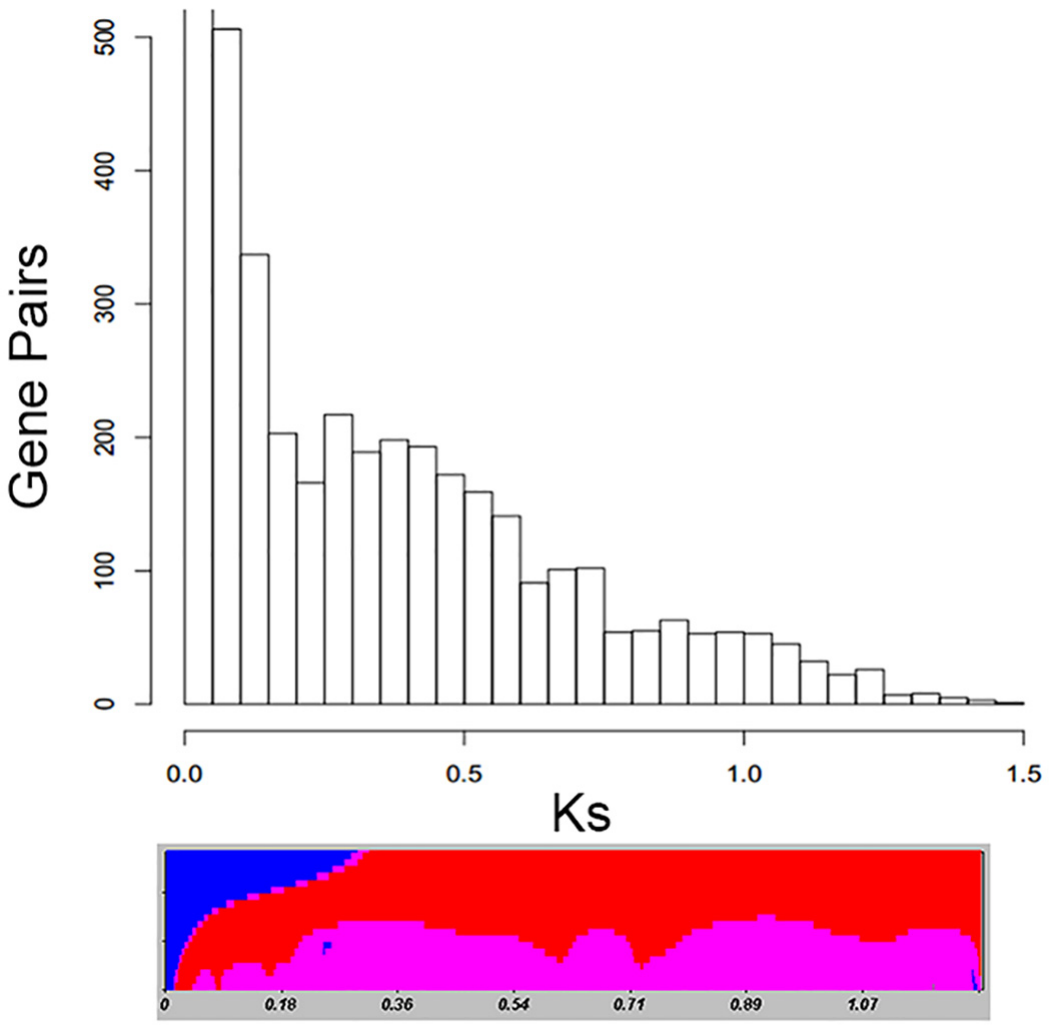
Histogram of KS values. A histogram of KS values with significant peaks identified in a SiZer graph below. Blue represents increases in slope; red indicates decreases; pink areas have no significant slope changes. A sharp increase at a KS ≈ 0.22 is indicated by a blue dot. This increase is followed by a broad pink peak of no changes with a decrease beginning at a KS of 0.60. Additional sharp declines are identified at KS of 0.71 and 1.34.

EMMIX detected seven peaks at Ks values of 0.01, 0.022, 0.05, 0.12 0.27, 0.51, and 0.91 (Fig 4). Peaks at Ks ≤ 0.1 represent current and most recent duplications. The remaining three peaks we considered as evidence for ancient duplications. From our initial analysis of significance using SiZer, only a single large peak from Ks ≈ 0.22 to 0.60 was shown to be significant. For comparison, we dated our duplications using the background mutation rate of 1.5 x 10^–8^ substitutions per synonymous site per year. We estimate these three duplication events that were in the predecessor of *A*. *arbuscula* ssp. *arbuscula* to be around 18 million years ago (mya), 34 mya and 60 mya.

## Discussion

We present three assembled transcriptomes of sagebrush now in the public domain of the Sequence Read Archive on NCBI (PRJNA258191, PRJNA258193, PRJNA258169). These transcriptomes add to the sparse amount of transcriptome data currently available for analysis in sagebrush. With a total of 61,883 transcripts identified by TRAPID, these transcriptome assemblies are a resource for advancing the characterization of species and subspecies and their chemical pathways related to defense, plant communication and a variety of other secondary compounds.

Sixteen transcripts with protein domains associated with terpene synthases (TSs) were identified, among them a putative Amorpha-4,11-diene synthase, the TS responsible for the popular and successful malaria drug artemisin. Terpenoids like artemisin comprise the largest groups of natural products with over 30,000 distinct chemical structures [13]. They are involved in a series of biological processes such as formation of biological structures, defense and signaling [26].

Many TSs have been found to synthesize multiple products from a single substrate [27], [28]. Thus, a single TS is of great importance in discovering and understanding a variety of terpenoid products. While chemical pathways radiating from a single TS to a diversity of terpenes have fundamentally been explained, the mechanism that switches between the different pathways is still unknown. Degenhardt et al. [29] assert that one of the best ways to improve understanding of TS function is to have more primary amino acid sequences in order to identify functional elements of TS. Transcripts allow for detection of protein functional groups that aid in detection of these elements. A putative ortholog TS of *M*. *chamomilla* was found in both *A*. *tridentata* and *A*. *arbuscula*. In chamomile, the orthologous TS is known as *MrTPS5* and has been found to produce mainly germacrene D, a volatile emission produced in response to herbivory [3]. However, demonstrating that TS produce multiple products, Irmish et al. [28] detected trace amounts of a variety of other terpenoids also produced by *MrTPS5*.

There were 38 SNPs between the chamomile transcript and the consensus sequence of *A*. *tridentata* and *A*. *arbuscula*—including 19 non-synonymous SNPs—which may contribute to a divergence of terpenoid products. Furthermore, the 6 bp deletion in sagebrush when compared to chamomile may indicate an autapomorphic feature derived within the tribe Anthemideae between the *Artemisia* and *Matricaria* genera. Whether these idiosyncrasies contribute to structural or functional differences in subsequent synthesis of terpenes has yet to be determined and deserves further attention.

The sequences of loci involved with terpene products could be important in classification and phylogenetic analysis because it has been shown that terpenes exist in different concentrations, ratios and types between species and subspecies of sagebrush [30], [31]. Exploiting these differences could bypass the subjective nature of morphology in favor of a genetic basis. This would be especially useful in the sagebrushes, where hybridization can make variable morphological characters difficult to interpret. The transcripts may prove more useful for defining phylogenies than their metabolic products because highly divergent TSs have been shown to produce the same product and highly similar TSs have been shown to produce different products [27], [32].

While our study focused primarily on TS transcripts, these transcriptomes possess a wealth of other research possibilities for studying sagebrush. For example, we also detected 39 transcripts related to the coumarin pathway. The coumarins are important for both the identification and ecological effect of sagebrush. Coumarins are a water-soluble class of chemicals that fluoresce blue when exposed to UV-light and vary in concentration in the different taxa of sagebrush [33]. Grinding sagebrush leaves in alcohol or water in the presence of UV-light can distinguish between different types of taxa such as *Artemisia arbuscula*—which fluoresces brightly—and *Artemisia tridentata* ssp. *wyomingensis*, which has little or no fluorescence. In addition, coumarin content can also predict the palatability of sagebrush; regardless of species, sage-grouse are proposed to select species of sagebrush with greater fluorescence [6]. These transcriptomes provide a genetic basis for this important chemical pathway.

One of the evolutionary forces that may contribute to chemical diversity within plants is polyploidy. Polyploidy is an evolutionary process that preserves genetic diversity, drives morphological complexity and may have afforded a greater resistance to extinction [34], [35]. At least one polyploid ancestor is suspected in all plant species [19]. These ancient duplications can be difficult to detect due to gene loss; however, analysis of existing paralogs can reveal a signal that lends to inference. In their study of ancient duplications in model plant species, Blanc and Wolfe [19] were unable to detect ancient duplications in any Asteraceae. Barker et al. [36] continued the work in Asteraceae from ESTs for 4 tribes of Asteraceae and found evidence for family level duplications in all samples as well tribe specific duplications in two samples. However, sequences for tribe Anthemidiae (which includes sagebrush) were not included in their study, and nearly all available sequences for genus *Artemisia* are from *A*. *annua*, a wormwood.

Our detection of ancient duplications revealed three secondary peaks with overlapping tails outside of the initial peak of recent gene duplications. The program SiZer was unable to differentiate two peaks identified by EMMIX and called a single broad peak from a Ks ≈ 0.22 to 0.60 as significant. We propose that the large overlap of these peaks obscures their identity. Evidence for two peaks is indicated by an additional sharp decline in the SiZer map at Ks = 0.71. Additional evidence for the peak at Ks = 0.51 is the replication of a similar peak by Barker et al. [36]. They were also unable to find the most ancient duplication (Ks = 0.91) as a significant peak using SiZer. However, our detection of this peak, as well as their detection of similar peaks in all four of their sampled tribes of Asteraceae makes us agree with their conclusion that the significance of this peak is obscured by the negative slope of more prominent recent duplications.

The presence of two of our secondary peaks is congruent with a study by Barker et al. [36] that demonstrated that tribes such as Cardueae and Cichorioideae within Asteraceae retain a detectable signal for the shared paleopolyploidization at Ks near 0.90, while others such as Mutisieae and Heliantheae possess signals for tribe specific paleopolyploidization events near a Ks of 0.50. Furthermore, based on their data they estimate that tribe-specific duplications should fall within the expected Ks range of 0.50–0.62; our detected Ks value of 0.51 falls within this range. We estimate a Ks value of 0.50 to correspond to 34 mya. This is near a previously estimated range (33–39 mya) for the radiation of the Asterodiae tribes [37], which includes the sagebrush tribe Anthemideae. The more ancient peak at Ks = 0.91 is likely an ancient paleopolyploidization shared by all members of the Asteraceae estimated 50 mya near the divergence of Asteraceae from its sister group Calyceraceae [37], [38].

The more recent peak at Ks = 0.27 corresponds to a time about 18 mya and was not detected in other tribes of Asteraceae sampled by Blanc and Wolfe [19] or Barker et al. [36]. This ancient duplication also occurred more recently than the estimates of Asteraceae tribe differentiation near a Ks value of 0.50. Instead, this peak at 18 mya may be evidence of a duplication event unique to the divergence of genus *Artemisia*. Similar results have been been reported using the most reliable pollen fossil of *Artemisia* for a calibration point and genetic data (nrDNA, ITS and ETS) by Sanz et al. [39]. They estimated the divergence of *Artemisia* from its most closely related genera to have taken place around 19.8 mya in the Early Miocene.

While it is not certain that these putative WGD resulted in these specific divergent events, Soltis et al. [40] have highlighted a positive correlation with the divergence of angiosperms in the recent aftermath of WGD. Furthermore, as we have shown, our estimated dates fall near other independently estimated dates for major events in the evolutionary history of sagebrush. This study lends genetic support to a divergence of the Asteraceae near 50 mya, the radiation of the Asterodieae tribes 33–39 mya and an ancient duplication event unique to genus *Artemisia* around 20 mya. This data also allows for future evolutionary and phylogenetic comparisons in the already described tribes of Asteraceae as well as more distantly related taxa.

## Conclusion

The three *Artemisia* assembled transcriptomes (*A*. *tridentata* ssp. *tridentata*, *A*. *tridentata* ssp. *wyomingensis* and *Artemisia arbuscula)* represent a significant increase not only in available sequence data for sagebrush, but also for genus *Artemisia* as a whole. These transcriptomes offer a foundation for future studies of sagebrush, including inferences in chemical defense and the identification of species and subspecies of sagebrush for restoration and preservation of the threatened sage-grouse. Our detection of ancient duplications also gives insight in the evolutionary history of sagebrush by providing evidence for three ancient duplications, one that is attributed to the divergence of Asteraceae, the other of the tribe Anthemidiae and the more recent duplication as unique to genus *Artemisia*.

## Supporting Information

S1 Table List of 16 transcripts associated with terpene synthases. Transcripts identified as being associated with terpene synthases by association with the HOM000066 gene family.
(DOCX)
