# Supplementary material for: Insights into Transcriptomes of Big and Low Sagebrush
Source: PLoS One. 2015 May 28;10(5):e0127593. doi: 10.1371/journal.pone.0127593 (PMC4447352; doi:10.1371/journal.pone.0127593)
Supplement: S1 Table — Transcripts identified as being associated with terpene synthases by association with the HOM000066 gene family. (DOCX) [file pone.0127593.s001.docx]

Supplementary 1

**Transcript Gene Family GO annotation InterPro annotation Subsets**

Ccav24930 568_HOM000066 GO:0000287,GO:0008152,GO:0016829, IPR001906,IPR008930,IPR005630, CAV,

Ccav26954 568_HOM000066 GO:0000287,GO:0008152,GO:0016829, IPR001906,IPR008930,IPR005630, CAV,

Ccav28248 568_HOM000066 GO:0000287,GO:0008152,GO:0016829, IPR001906,IPR008930,IPR005630, CAV,

Ccav28334 568_HOM000066 GO:0000287,GO:0008152,GO:0016829, IPR001906,IPR008930,IPR005630, CAV,

Ccav44821 568_HOM000066 GO:0000287,GO:0008152,GO:0016829, IPR001906,IPR008930,IPR005630, CAV,

Ccav49365 568_HOM000066 GO:0000287,GO:0008152,GO:0016829, IPR001906,IPR008930,IPR005630, CAV,

Ccav52791 568_HOM000066 GO:0000287,GO:0008152,GO:0016829, IPR001906,IPR008930,IPR005630, CAV,

Ccav56438 568_HOM000066 GO:0000287,GO:0008152,GO:0016829, IPR001906,IPR008930,IPR005630, CAV,

Ccav60306 568_HOM000066 GO:0000287,GO:0008152,GO:0016829, IPR001906,IPR008930,IPR005630, CAV,

Ccav62982 568_HOM000066 GO:0000287,GO:0008152,GO:0016829, IPR001906,IPR008930,IPR005630, CAV,

Ccav76367 568_HOM000066 GO:0000287,GO:0008152,GO:0016829, IPR001906,IPR008930,IPR005630, CAV,

Ccav80259 568_HOM000066 GO:0000287,GO:0008152,GO:0016829, IPR001906,IPR008930,IPR005630, CAV,

Cutt12295 568_HOM000066 GO:0000287,GO:0008152,GO:0016829, IPR001906,IPR008930,IPR005630, UTT2,

Cutt25863 568_HOM000066 GO:0000287,GO:0008152,GO:0016829, IPR001906,IPR008930,IPR005630, UTT2,

Cutt5751 568_HOM000066 GO:0000287,GO:0008152,GO:0016829, IPR001906,IPR008930,IPR005630, UTT2,

Cutw15546 568_HOM000066 GO:0000287,GO:0008152,GO:0016829, IPR001906,IPR008930,IPR005630, UTW1,

Supplementary 1 List of transcripts with a protein domain associated with terpene synthases.
